# 719. *Stenotrophomonas maltophilia* in Japanese hospitals: Clinical characteristics and molecular epidemiology of infection and colonization cases registered in multicenter surveillance network

**DOI:** 10.1093/ofid/ofad500.781

**Published:** 2023-11-27

**Authors:** Ryota Hase, Aki Sakurai, Masahiro Suzuki, Naoya Itoh, Sho Saito, Kayoko Hayakawa, Kohei Uemura, Yasufumi Matsumara, Hideaki Kato, David van Duin, Norio Ohmagari, Yohei Doi

**Affiliations:** Japanese Red Cross Narita Hospital, Narita-shi, Chiba, Japan; University of Texas Health Science Center, McGovern Medical School, Houston, TX; Fujita Health University School of Medicine, Toyoake, Aichi, Japan; Aichi Cancer Center, Nagoya, Aichi, Japan; National Center for Global Health and Medicine, Shinjuku-ku, Tokyo, Japan; National Center for Global Health and Medicine, Shinjuku-ku, Tokyo, Japan; The University of Tokyo, Bunkyo-ku, Tokyo, Japan; Kyoto University Graduate School of Medicine, Kyoto, Kyoto, Japan; Yokohama City University Hospital, yokohama-shi, Kanagawa, Japan; University of North Carolina at Chapel Hill, Chapel Hill, NC; National Centre for Global Health and Medicine, Shinjuku, Tokyo, Japan; Fujita Health University School of Medicine, Toyoake, Aichi, Japan

## Abstract

**Background:**

*Stenotrophomonas maltophilia* has become one of the major gram-negative pathogens causing nosocomial infections. However, comprehensive analysis of the clinical characteristics and molecular epidemiology of patients with *S. maltophilia* remains limited.

**Methods:**

All patients with a clinical culture growing *S. maltophilia* were collected from April 2019 to March 2022 through the Multi-Drug Resistant organisms clinical research network (MDRnet), consisting of 12 tertiary care hospitals in Japan. The clinical characteristics, outcomes, antimicrobial susceptibility and molecular epidemiology of cases with *S. maltophilia* colonization and infection were investigated and compared.

**Results:**

In total, 78 cases, 44 with colonization and 34 with infection, were included. The median age was 72.5 years (IQR: 61–78), and males accounted for 53 cases (67.9%). The most common comorbidity was localized solid malignancy (38.5%). Almost half of the patients (43.6%) were immunosuppressed, and the most common reason was antineoplastic chemotherapy (30.8%). Respiratory tract was the most common site of colonization (86.4%), whereas bacteremia accounted for more than half of infection cases (55.9%). The overall 30-day all-cause mortality rate was 20.5%, which was significantly higher in infection cases than in colonization cases (35.3% vs 9.1%; odds ratio, 5.33; 95% confidence interval, 1.40–25.45). Susceptibility rates to ceftazidime, levofloxacin, minocycline, and sulfamethoxazole-trimethoprim were 14.1%, 65.2%, 87.2%, and 100%, respectively. Multilocus sequence typing of the 78 strains revealed that they belonged to 62 different sequence types (STs), of which 42 were known STs and 36 were novel STs.

Kaplan-Meier survival analysis

**Figure:**
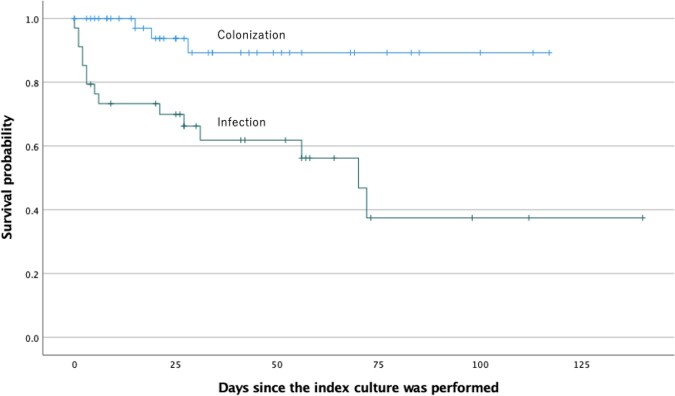

**Conclusion:**

*S. maltophilia* frequently colonizes the respiratory tract, and the mortality is significantly higher in infection cases. Sulfamethoxazole-trimethoprim remains active, but susceptibility to levofloxacin appear to be declining. The strains were genetically highly diverse, consistent with the likely environmental origin.

**Disclosures:**

**Masahiro Suzuki, PhD**, KANTO Chemical co., inc.: Grant/Research Support **Sho Saito, MD, PhD**, Shionogi & Company, Limited: Grant/Research Support **Yasufumi Matsumara, MD, PhD**, Beckman Coulter: Grant/Research Support|Presicion System Science: Grant/Research Support|Toyobo: Grant/Research Support **David van Duin, MD, PhD**, Entasis: Advisor/Consultant|Merck: Advisor/Consultant|Merck: Grant/Research Support|Pfizer: Advisor/Consultant|Pfizer: Honoraria|Qpex: Advisor/Consultant|Roche: Advisor/Consultant|Shionogi: Advisor/Consultant|Shionogi: Grant/Research Support|Union: Advisor/Consultant|Utility: Advisor/Consultant **Yohei Doi, MD, PhD**, bioMerieux: Advisor/Consultant|FujiFilm: Advisor/Consultant|Gilead: Advisor/Consultant|Gilead: Honoraria|GSK: Advisor/Consultant|Meiji Seika Pharma: Advisor/Consultant|Moderna: Advisor/Consultant|Moderna: Honoraria|MSD: Advisor/Consultant|MSD: Honoraria|Shionogi: Advisor/Consultant|Shionogi: Grant/Research Support|Shionogi: Honoraria

